# Successful treatment of acquired reactive perforating collagenosis with itraconazole

**DOI:** 10.1186/s40001-021-00542-6

**Published:** 2021-07-13

**Authors:** Binrong Ye, Yi Cao, Yeqiang Liu

**Affiliations:** 1 Zhejiang Chinese Medical University, No. 548, Binwen Road, Binjiang district, Hangzhou, 310053 Zhejiang China; 2 Dermatology, Suzhou TCM Hospital Affiliated to Nanjing University of Chinese Medicine, No.18 Yang Su Road, Suzhou, 215009 Jiangsu China; 3 Dermatopathology, Shanghai Skin Disease Hospital, No. 1278 Baode Road, Zhabei District, Shanghai, 200443 China

**Keywords:** Itraconazole, Acquired reactive perforating collagenosis, Anti-inflammatory, Anti-angiogenic

## Abstract

**Background:**

Acquired reactive perforating collagenosis (ARPC) is a rare form of transepithelial elimination in which altered collagen is extruded through the epidermis.

**Case presentation:**

A 23-year-old male presented with cup-like ulcerated lesions on his limbs since 3 months. A series of serological and immunological tests showed no abnormalities. A diagnosis of ARPC was based on skin biopsy findings. The patient was cured using treatment with itraconazole for 8 weeks, in the absence of a fungal infection.

**Conclusions:**

The anti-inflammatory and anti-angiogenic effects of itraconazole can have good therapeutic benefits for ARPC.

## Background

Acquired reactive perforating collagenosis (ARPC) is an underdiagnosed dermatosis of unknown aetiology and pathogenesis and is frequently associated with systemic disorders [1]. It is a rare form of classical perforating dermatosis, which is classified into reactive perforating collagenosis (RPC), elastosis perforans serpiginosa, perforating folliculitis, and Kyrle disease [1]. It often manifests as umbilicated hyperkeratotic papules or a dome-shaped lesion with a central crater, which is more common in the extremities. It is characterised by transepidermal elimination of altered collagen fibres. Diagnosis is established by the clinical presentation and characteristic histopathological findings. According to the following criteria, of transepidermal elimination of basophilic collagen bundles, central hyperkeratotic or cup-like depressed papules, and onset after 18 years old. The treatment of ARPC involves controlling the underlying disease and relieving itching. However, the treatment consensus for ARPC is not clear, and reported therapies are diverse [2]. Herein, we present a rare case of ARPC without systemic disease, which was effectively treated with itraconazole.

## Case presentation

A 23-year-old man consulted our department for skin ulcers on his extremities that persisted for more than 2 months, with mild itching. The patient reportedly remained healthy until he developed tinea pedis infection in the left foot more than 2 months ago. Improvement was noted after topical application of terbinafine hydrochloride ointment and furacillin solution at a dermatological hospital. However, following the itching phase, symptoms of urticaria developed rapidly. Symptomatic relief was achieved following a 3-day treatment with oral glucocorticoids and antihistamines. After a few days, round superficial ulcers began to appear linearly in the lower limbs, and gradually spread to the upper limbs; however, the itching was not present this time. The patient also denied that the skin lesion was formed due to scratching.

**Fig. 1:**
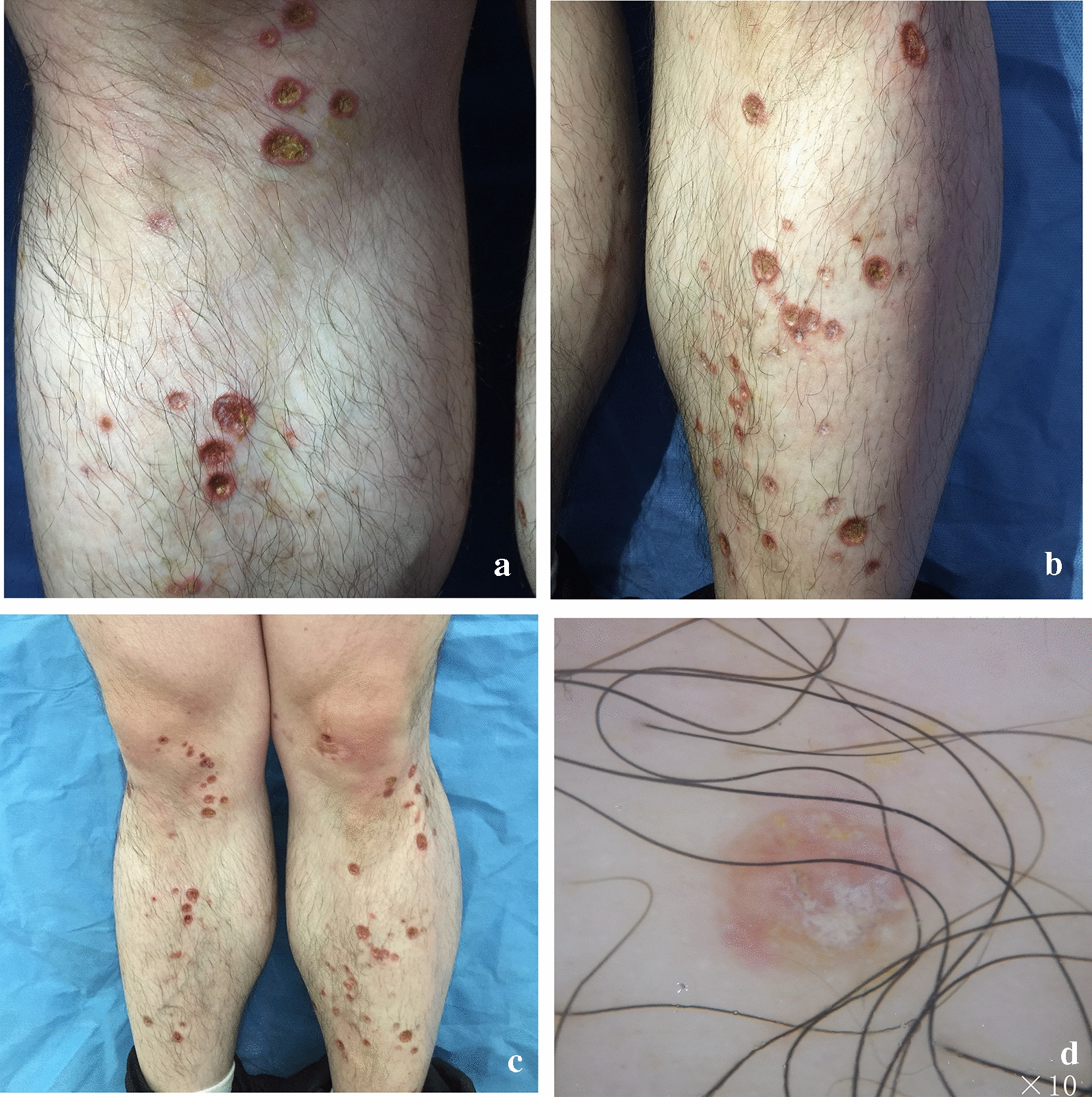
**a**–**c** Physical examination revealing shallow cup-shaped ulcers in the extremities and a linear distribution of the skin lesions. **d** Dermoscope revealed a rounded ulcer covered with yellow crusts at fifth week

The differential diagnoses in this case were sporotrichosis, syphilis, vasculitis, perforating dermatosis, *Talaromyces marneffei* infection, dermatitis artefacta, and drug rash. Physical examination revealed shallow cup-shaped ulcers in the extremities and a linear distribution of the skin lesions (Fig. 1). Koebner phenomenon and initial fungal microscopic examination findings of the skin of the feet and lower limbs were negative. The results of a series of serological tests, including acquired immunodeficiency syndrome screening, treponema pallidum particle agglutination assay, rapid plasma reagin test, liver and kidney function tests, blood glucose analysis, whole blood cell test, and erythrocyte sedimentation, C-reactive protein, anti-streptolysin “O”, antinuclear antibody, extractable nuclear antigen, and antineutrophil cytoplasmic antibody tests, were negative. Mycobacterial culture and identification were negative, and no fungus was detected. A skin biopsy was subsequently scheduled, and the patient was suggested a diagnostic treatment with itraconazole. Considering the possibility of a fungal infection due to tinea pedis infection at the early stages of onset, we administered oral itraconazole 200 mg BID as a therapeutic challenge. Unexpectedly, the ulcers rapidly healed and formed scars in a week.

**Fig. 2:**
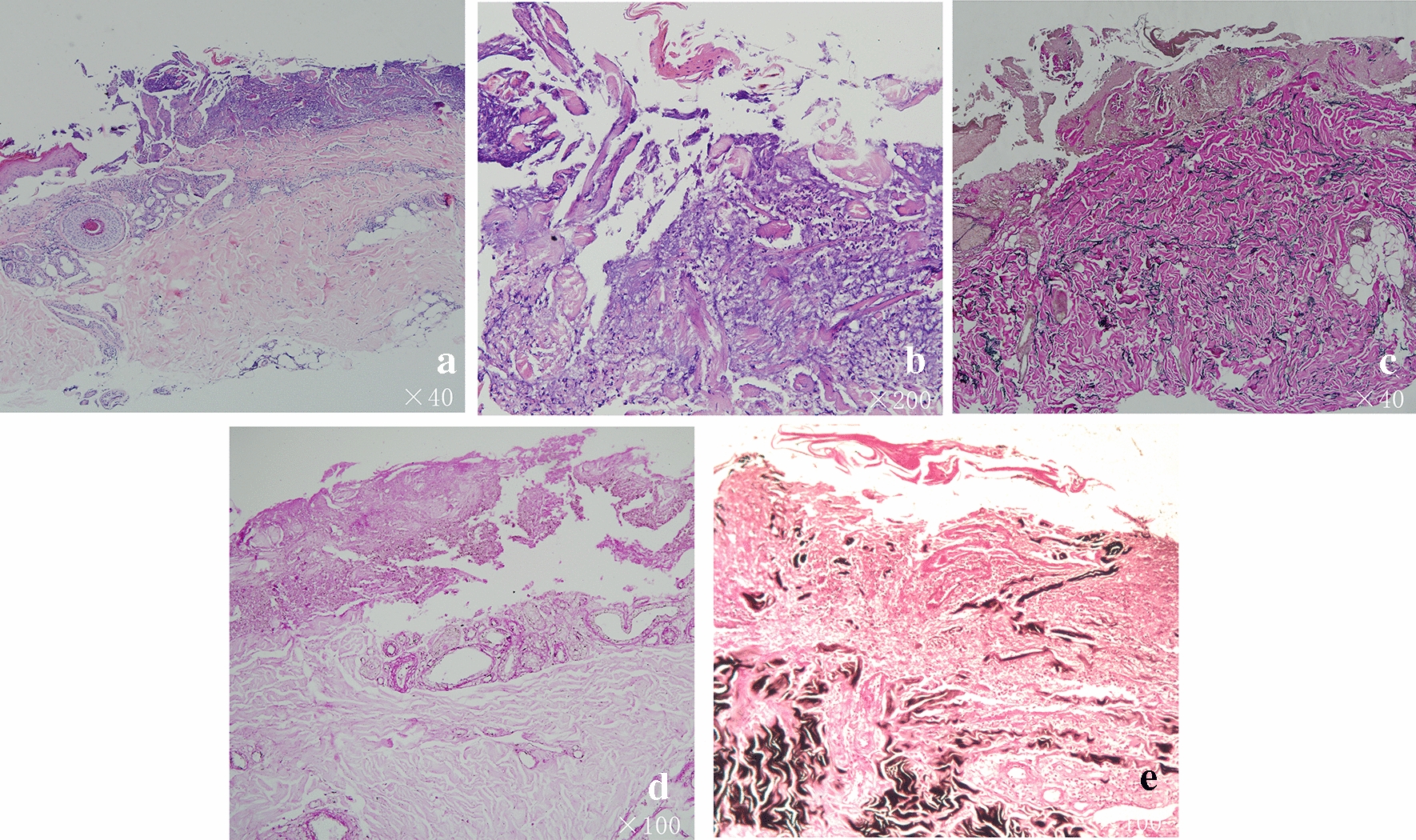
**a** Lesional skin biopsy showing a cup-shaped depression plugged with necrotic inflammatory debris (haematoxylin and eosin staining [H&E] × 40). **b** The central crusted keratotic plug contains keratin, cellular debris, and fibrous tissue (H&E × 200). **c** van Gieson staining (original magnification  ×40) showing elimination of fibrous tissue through the dermis into the epidermis. **d** No fungal hyphae were found on periodic acid–Schiff staining (original magnification ×100). **e** Gomori’s methenamine silver staining shows the transepidermal fibres and the absence of fungi (original magnification ×100)

**Fig. 3:**
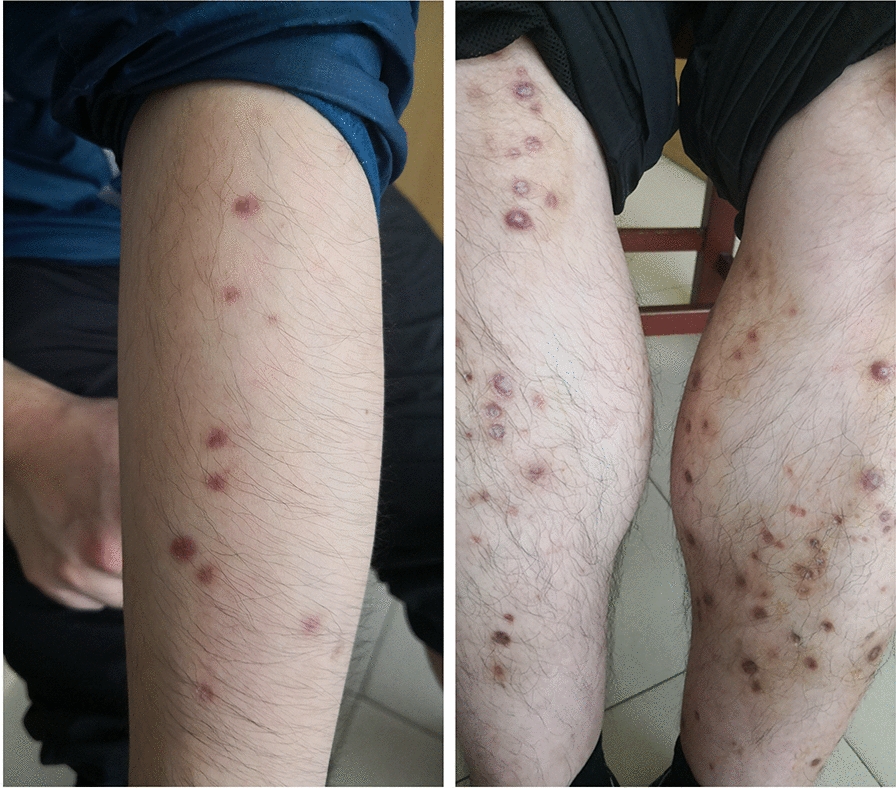
Results after 8 weeks of treatment

At this time, the results of haematoxylin and eosin staining indicated perforating dermatosis. On elastica van Gieson staining, the fibrous tissue was found ejected through the epidermis (Fig. 2). In correlation with the clinical findings, a diagnosis of ARPC was considered. To determine the aetiology and an evidence-based basis for treatment, hexamine Gomori’s methenamine silver staining and periodic acid–Schiff staining were performed, but no evidence of a rare fungal infection was found. In the fourth week, itraconazole treatment was discontinued, but the patient’s condition worsened after interruption. On dermoscopy (DL4D2035), the ulceration reappeared (Fig. 1d). With the consent of the ethics committee and the patient, itraconazole 200 mg BID for 8 weeks was administered. Following this, while the ulcers did not recur, multiple scar formation was observed (Fig. 3).

## Discussion

This is a case of atypical ARPC without an associated disease. The pathogenesis of this disease is not exactly the same as that of classic ARPC, which has led to some people considering atypical ARPC to be pseudoperforation, often triggered by scratching or superficial trauma. However, in our case, the skin lesions were symmetric and crater-like, and the ARPC diagnosis was clinically and pathologically indisputable. Although some drugs were administered before disease onset, there is no evidence that the condition was drug-induced. Some ARPC cases are associated with complications such as diabetes mellitus, chronic renal disease, AIDS, pulmonary fibrosis, tumours, thyroid disease, liver failure, scabies, and insect bites [3]. Interestingly, two cases did not show laboratory abnormalities. The present case is also rare. Regular medical examinations for 2 consecutive years have confirmed that the patient is healthy. We suspect that the disease is the result of a series of immune responses triggered by tinea pedis.

While several modes of treatment have been investigated, there is no effective standard treatment of ARPC. Allopurinol therapy has been effective in treating ARPC, but only for patients with diabetes mellitus [4, 5]. Thus, it is worth exploring how the target therapy for treating ARPC should be selected. Collagen constitutes one of the vital components of the basement membrane scaffolds. Non-collagenous domains derived from collagen attaining significance in regulation of angiogenesis promoted diseases. The collagen eliminated in RPC may be derived from the basement membrane zone [6]. Angiogenesis is a crucial mechanism of vascular growth and regeneration that requires biosynthesis and cross-linking of collagen in vivo and is induced by collagen in vitro [7]. The anti-angiogenic, anti-Hedgehog pathway, and anti-inflammatory properties of itraconazole have been widely reported [6]. The effectiveness of treatment of lichen planus, mycosis fungoides, basal cell carcinoma, palmoplantar pustulosis, and infantile haemangioma has been reported [8]. Furthermore, we found that itraconazole has a significant effect on ARPC whose pathogenesis may involve vascular damage [1]. Thus, we infer that the possible mechanism was based on the inhibition of fibroblast growth factors and vascular endothelial growth factors to treat ARPC [9]. In particular, itraconazole inhibits neutrophil chemotaxis, interleukin-8 production, and the formation of pro-inflammatory metabolites, which are beneficial for ulcer healing [10].

## Conclusions

To the best of our knowledge, this is the first time itraconazole has been used to treat ARPC, and it could be used to treat other types of ARPC through a common mechanism of action, such as vascular damage; however, further studies are needed to confirm this. Nonetheless, the side effects and cost of 200 mg itraconazole BID for > 4 weeks should be carefully considered. We believe that this study will contribute to the literature on ARPC pathogenesis and treatment.

## Data Availability

The data that support the findings of this study are available from the corresponding author upon reasonable request.

## Abbreviations

ARPC: Acquired reactive perforating collagenosis
RPC: Reactive perforating collagenosis
